# A Retrospective Study of Virtual Hospital Experiential Learning Across Clerkship and Pre-clerkship Doctor of Osteopathic Medicine Students at a Medical School Based in the United States

**DOI:** 10.7759/cureus.72115

**Published:** 2024-10-22

**Authors:** Lydia Kersh, Anthony Sciuva, Jennifer Shaw

**Affiliations:** 1 General Surgery, Philadelphia College of Osteopathic Medicine, Philadelphia, USA; 2 General Surgery, Philadelphia College of Osteopathic Medicine South Georgia, Moultrie, USA; 3 Biomedical Sciences, Philadelphia College of Osteopathic Medicine South Georgia, Moultrie, USA

**Keywords:** clinical skills, medical education, medical simulation, perceived confidence, virtual clerkship, virtual hospital, virtual simulation

## Abstract

Introduction: During the COVID-19 pandemic, a medical institution in the United States implemented a virtual clerkship rotation to replicate the skills that students would develop during an in-person clinical site rotation. The primary objective of this study was to identify the efficacy and benefits following the implementation of the Virtual Hospital Experiential Learning (VHEL) model, as demonstrated by increased perceived confidence and skill growth among medical students.

Methods: Cases were presented to five to six medical students each week. On the Monday of the week, students entered a virtual conference platform for a standardized patient encounter. Staff and faculty randomly assigned all students to sessions. A post-VHEL survey was conducted to determine the level of perceived student confidence (measured via the Likert scale, self-reported).

Results: From the 193 students randomly assigned to VHEL sessions, 49 survey responses were gathered, resulting in a 25% response rate. According to our dataset, one week following the VHEL sessions, students reported a significant increase in their perceived confidence regarding diagnostic skills and problem-solving within a clinical setting. The majority of respondents felt that they had gained confidence and skills through the sessions. Additionally, they noted that the sessions effectively mirrored the experiences of in-person clerkships in a hospital environment.

Conclusion: Most medical student respondents reported an increase in perceived overall confidence following the completion of the VHEL sessions. First-year medical student data displayed a moderately higher level of perceived total confidence overall, suggesting that the implementation of VHEL sessions during the spring semester of the first year of medical school may offer the most significant gains.

## Introduction

Coronavirus disease 2019 (COVID-19) was a novel virus discovered in late 2019. In most individuals, COVID-19 will cause respiratory illness with a full recovery, while in some, it may lead to severe breathing problems and death [1]. This disease quickly spread throughout the world and led to a pandemic that forced many countries to enter a lockdown. On March 23rd, 2020, the city of Philadelphia enforced stay-at-home orders, and the institutions in the city were required to operate remotely to minimize any activities in person [2]. This mandate greatly affected medical schools in the area and throughout the world. A medical institution in the United States with three campuses transferred all learning to a remote platform on March 16th, 2020. Teaching labs were postponed by the medical institution and access to clinical clerkships was dictated by the hospitals wherein they were situated; the medical institution’s staff were directed to work remotely, and large gatherings were canceled [3]. Medical students working in the hospitals were required to pivot to a remote learning style rather than a primary hands-on mode of learning with patients. This was particularly challenging for third-year (M3) and fourth-year (M4) medical students during their essential phase of clinical education in preparation for residency.

Medical students were the first trainees to be removed from the hospital at the onset of the pandemic due to personal protection equipment (PPE) and coronavirus testing kits shortages, a result of supply prioritization for essential workers. Moreover, hospitals did not want to endanger their trainees. Displacement from the hospitals limited live, real-time feedback for students, a crucial step in their development as clinicians. During the initial period of the pandemic, an eye hospital in Hong Kong reported its approach to overcoming this limitation by educating trainees on procedures and clinical cases via virtual platforms. The trainees observed simulated procedures and then performed them under supervision [4].

The medical institution sought to simulate the clinical experiences M3 and M4 medical students would traditionally learn in an in-person hospital setting; therefore, the school developed Virtual Hospital Experiential Learning (VHEL) sessions as a required substitute for hospital rotations. A systematic review by Dedeilia et al. demonstrated that virtual video conferences and simulations, such as VHEL sessions, were the optimal approaches to replace the traditional in-person learning experience [4]. Furthermore, a clinical study by De Ponti et al. reported students embraced virtual simulation of hospital experiences [5]. While it was not ideal to transform education modalities for medical students and faculty on such short notice, the pilot VHEL sessions initiated at the medical institution were a viable alternative to in-person clinical scenarios utilizing a similar approach successfully implemented by other medical schools early in the pandemic [4,5].

The VHEL model provided a resource for students to practice clinical skills while removed from their training hospitals. Medical educators at this institution, along with others across the globe, rapidly adjusted curricular delivery approaches out of necessity and worked creatively to care for and engage students during a unique and stressful period. The post-pandemic years have enabled reflection on the educational tools employed, thus providing an opportunity to gain insight into the approaches to retain moving forward. As such, we present a retrospective analysis of the virtual hospital student experience during clerkship (M3-M4) and pre-clerkship (M1-M2) years of medical education. The study analyzes student survey response data to ascertain self-reported confidence in decision-making and skills toward patient care and treatment following VHEL sessions. These data contribute to the growing body of literature informing curricular design toward effective delivery of medical education in response to the post-pandemic evolution of student learning styles, preferences, and modalities.

## Materials and methods

Virtual Hospital Experiential Learning (VHEL) sessions

The medical institution’s VHEL sessions involved case presentations to groups of five to six M3 and M4 medical students each week for a total of six weeks. Using a virtual online meeting platform, students met each Monday for a standardized patient encounter. The students collected a history based on a preset clinical presentation and diagnosis. They were instructed to write a hospital admission note for submission via the institution's learning management system (LMS), with all requested labs sent via email to the faculty. Faculty submitted the patient’s lab results via LMS and students responded to any “emergency” calls for the week regarding the patient. Students submitted further lab orders and instructions for patient care to faculty via LMS. On Wednesdays of each week, students met virtually with a faculty member regarding a second patient, and students collected the history and clinical presentation of the second patient. Based on the clinical presentation, students decided whether to admit the patient to the virtual hospital. Students were on call throughout the week for both patients. By Friday of the same week, students indicated to faculty whether they would discharge the patients or keep them admitted to the virtual hospital. On that same day, the students who participated in the VHEL session met with faculty for feedback on their weekly performance in caring for the patient and the logistics of properly conducting oneself as a physician in a hospital setting. Due to the high student and faculty engagement level in the required sessions, VHEL sessions were offered as an elective during the pandemic for first-year (M1) and second-year (M2) students to practice and gain confidence in clinical skills in preparation for their future clerkships.

Description of survey response dataset

Survey response data utilized in this retrospective study were collected in 2022 from medical students enrolled between 03/2020 and 05/2022 at one of the three campuses of a medical institution within the US. There were 49 respondents from the 193 students randomly assigned to the VHEL sessions (25% response rate). Pre-clerkship medical student (M1 and M2) respondents were enrolled in the VHEL sessions as an elective course, while clerkship medical students (M3 and M4) respondents were enrolled in VHEL sessions as a requirement of their curriculum. Outcome measures were variables that may be assessed independent of class year (M1-M4) or previous experience. Responses to statements provided measurable and quantifiable data to reveal changes in students' perceived total confidence in their clinical skills following VHEL.

Data analyses

This retrospective study analyzed Likert scale data collected following VHEL sessions offered during the pandemic. Student response data were based on the survey statements listed in Table 1. Students responded whether they strongly disagree, disagree, neither disagree nor agree, agree, or strongly agree with each statement. Four or more Likert-type items representing similar statements were combined to produce a single composite score of perceived total confidence. Principal components factor analysis (PCA) was used to group the items into the subscale of perceived total confidence. The composite score resulted from the sum of responses to statements 3, 4, 6, 8, 9, 12, and 14 (Table 1). Likert scale score data were analyzed as interval data and means and standard deviations were utilized to describe the scale and calculate standard error. Perceived total confidence scores across medical school years were plotted with standard error, and the percent total response for each question was calculated using RStudio (Posit, Boston, MA). A two-sample t-test was conducted to compare pre-clerkship students (M1 and M2) and clerkship students (M3 and M4) using IBM's SPSS version 29 (IBM Corp., Armonk, NY) to investigate any relationship between the confidence scores and year in medical school. A linear regression analysis of survey responses and years in medical school was conducted using the statistical software RStudio [6]. A qualitative analysis of the survey statement responses was conducted to investigate students' perceptions of the VHEL sessions.

**Table 1:** REDCap post-VHEL survey statements and response percent. Respondents were instructed to select across a 1-5 Likert scale. VHEL: Virtual Hospital Experiential Learning; CRIBS: Clinical Reasoning in Basic Sciences. REDCap (Vanderbilt University, Nashville, TN).

Survey statements	Likert scale responses	Number of respondents (n = 41)
1. I learned something new during the VHEL sessions.	Strongly disagree	1 (2.4%)
	Agree	14 (34%)
	Strongly agree	26 (63%)
2. The VHEL sessions are what I expect to encounter when I begin rotations.	Disagree	2 (4.9%)
	Neither disagree nor agree	11 (27%)
	Agree	16 (39%)
	Strongly agree	12 (29%)
3. The VHEL sessions increased my confidence when preparing and presenting a case for CRIBS.	Strongly disagree	1 (2.4%)
	Disagree	2 (4.9%)
	Neither disagree nor agree	9 (22%)
	Agree	17 (41%)
	Strongly agree	12 (29%)
4. I am better prepared to formulate a working diagnosis and differentials after participating in the VHEL sessions.	Strongly disagree	1 (2.4%)
	Neither disagree nor agree	5 (12%)
	Agree	19 (46%)
	Strongly agree	16 (39%)
5. I learned how to write an admission note during the VHEL sessions.	Strongly disagree	1 (2.4%)
	Disagree	1 (2.4%)
	Neither disagree nor agree	14 (34%)
	Agree	16 (39%)
	Strongly agree	9 (22%)
6. I feel less hesitant to speak up in front of others and offer insight after participating in VHEL sessions.	Strongly disagree	1 (2.4%)
	Disagree	3 (7.3%)
	Neither disagree nor agree	12 (29%)
	Agree	12 (29%)
	Strongly agree	13 (32%)
7. I was worried about harming the patient during the VHEL sessions.	Strongly disagree	3 (7.3%)
	Disagree	13 (32%)
	Neither disagree nor agree	12 (29%)
	Agree	8 (29%)
	Strongly agree	5 (12%)
8. I became more decisive in my clinical decision-making throughout the week of VHEL sessions.	Strongly disagree	1 (2.4%)
	Disagree	1 (2.4%)
	Neither disagree nor agree	7 (17%)
	Agree	20 (49%)
	Strongly agree	12 (29%)
9. As a result of the VHEL sessions, I am more prepared and confident in conversing with and deducing the condition of standardized patients in my primary skills course.	Strongly disagree	1 (2.4%)
	Neither disagree nor agree	9 (22%)
	Agree	18 (44%)
	Strongly agree	13 (32%)
10. After this exercise, I researched and reviewed content related to patients in the VHEL sessions.	Strongly disagree	2 (4.9%)
	Disagree	1 (2.4%)
	Neither disagree nor agree	4 (9.8%)
	Agree	23 (56%)
	Strongly agree	11 (27%)
11. I am able to prioritize critical issues more efficiently in terms of patient treatment after participation in VHEL sessions.	Strongly disagree	1 (2.4%)
	Neither disagree nor agree	3 (7.3%)
	Agree	27 (66%)
	Strongly agree	10 (24%)
12. I am able to interact with patients in a critical situation more effectively after participating in VHEL sessions.	Strongly disagree	1 (2.4%)
	Disagree	1 (2.4%)
	Neither disagree nor agree	10 (24%)
	Agree	22 (54%)
	Strongly agree	7 (17%)
13. I am able to transcribe notes more accurately after participation in the VHEL sessions.	Strongly disagree	1 (2.4%)
	Disagree	1 (2.4%)
	Neither disagree nor agree	14 (34%)
	Agree	19 (46%)
	Strongly agree	6(15%)
14. I think the VHEL sessions improved my ability to order appropriate labs specific to patient treatment needs.	Strongly disagree	1 (2.4%)
	Neither disagree nor agree	7 (17%)
	Agree	22 (54%)
	Strongly agree	11 (27%)
15. The VHEL session is translatable to rotations.	Strongly disagree	1 (2.4%)
	Disagree	1 (2.4%)
	Neither disagree nor agree	7 (17%)
	Agree	20 (49%)
	Strongly agree	12 (29%)

## Results

**Figure 1:**
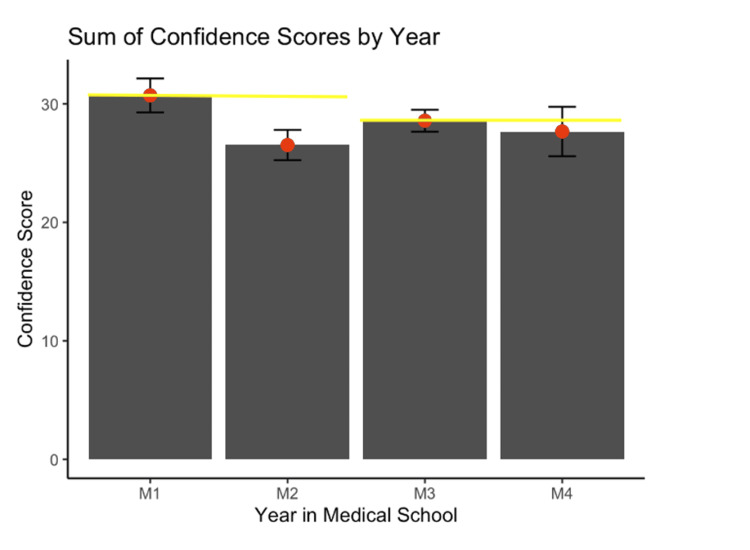
Perceived confidence scores of medical students by year in medical school after completing the VHEL session. Mean composite confidence scores and standard error between pre-clerkship (M1-M2) respondent data and clerkship (M3-M4) respondent data were analyzed using a two-sided t-test assuming equal variance (M1-M2, p = 0.09; M3-M4, p = 0.68). VHEL: Virtual Hospital Experiential Learning.

To elucidate the impact of VHEL sessions on perceived total confidence in clinical skills, response data were analyzed across years in medical school. A perceived total confidence score of 35 indicates the participant ranked all confidence-related statements as strongly agreed, wherein each statement is worth a numeric score of 5; in contrast, a score of 7 indicates the participant ranked all confidence-related statements as strongly disagreed, wherein each statement is worth a numeric score of 1 (Figure 1). The mean score and standard error (SE) were calculated for students spanning four years in medical school. The mean of M1’s perceived total confidence composite score was 30.71 (SE of 1.430), while the mean of M2’s perceived total confidence composite score was 26.52 (SE of 1.28). For the clerkship students, the mean of M3’s perceived total confidence composite score was 28.57 (SE of 0.92), and M4’s was 27.67 with a (SE of 2.08), as shown in Figure 1.

A primary analysis of the data was performed via a two-sample t-test to compare perceived total confidence levels between pre-clerkship (M1 and M2) students. There was no statistically significant difference between the M1 and M2 groups (p = 0.09) (Figure 1). A two-sample t-test to compare perceived total confidence levels between clerkship (M3 and M4) students showed no statistically significant difference between the M3 and M4 groups (p = 0.68), as shown in Figure 1. Linear regression was utilized to predict any change occurring across the continuum of years in medical school (M1-M4) in relation to the 1-5 Likert scale of responses to the survey statements. The β coefficient of -0.47 indicated each additional year in medical school will decrease the Likert response (1-5) by 0.47 units on average, which was found to not be statistically significant (p = 0.6, Table 2).

**Table 2:** Linear regression analysis to determine the relationship between the independent variable response to the statement based on the 1-5 Likert scale and the dependent variable medical school year. A p-value < 0.05 was considered significant. 1 = strongly disagree, 2 = disagree, 3 = neither disagree nor agree, 4 = agree, and 5 = strongly agree.

Characteristic	Beta	95% confidence interval	p-value
Year	-0.47	-2.2, 1.3	0.6

Theme 1: Overall perception of the sessions

Medical students were asked to agree or disagree (across a 1-5 Likert scale) with statements that represented the overall experience of the VHEL sessions. When responding to the following statement, “I learned something new during the VHEL sessions,” 97% of respondents agreed or strongly agreed, indicating participants' positive experience with the session. Students also responded to the statement, “The VHEL sessions are what I expect to encounter when I begin rotations,” and 68% of respondents agreed or strongly agreed. The majority of respondents felt the session was conducted as expected. Students were also presented with the statement, “After this exercise, I researched and reviewed content related to patients in the VHEL sessions,” and 83% of respondents agreed or strongly agreed, indicating respondents were engaged enough with the material, that on their own time, they initiated research of the topics when neither required nor asked to do so. When responding to the following statement, “The VHEL session is translatable to rotations,” 78% of respondents agreed or strongly agreed, suggesting they perceived the session mimicked the skills used and gained while on in-person clerkship rotations (Table 2).

Theme 2: Perceived skills gained

Medical students were asked to agree or disagree (across a 1-5 Likert scale) with statements that represented the perceived skills gained during the VHEL sessions. When responding to the following statement, “I am better prepared to formulate a working diagnosis and differentials after participating in the VHEL sessions,” 85% of respondents agreed or strongly agreed. This emphasizes the respondents' belief they improved this skill set of clinical reasoning. When responding to the following statement, “I learned how to write an admissions note during the VHEL sessions,” 61% of respondents agreed or strongly agreed. The majority of respondents believed they developed the ability to write an admission note, a skill integral to clerkship and beyond. When responding to the following statement, “I am able to prioritize critical issues more efficiently in terms of patient treatment after participation in VHEL sessions,” 90% of respondents agreed or strongly agreed. Based on that percentage of responses, almost all students believed they could critically evaluate and effectively treat a patient better after the session. Furthermore, 71% of respondents agreed or strongly agreed with the statement, “I am able to interact with patients in a critical situation more effectively after participation in VHEL sessions,” suggesting improved critical thinking skills with patients after the session. When responding to the following statement, “I am able to transcribe notes more accurately after participation in the VHEL sessions,” 61% of respondents agreed or strongly agreed. This indicates the majority of respondents felt they increased their attention to detail during patient encounters after participation in VHEL sessions, a skill relevant at all levels of medical education. When responding to the following statement, “I think the VHEL sessions improved my ability to order appropriate labs specific to patient treatment needs,” 81% of respondents agreed or strongly agreed, suggesting an enhancement of clinical reasoning and decision-making while ordering labs.

Theme 3: Perceived confidence

Medical students were asked to agree or disagree (across a 1-5 Likert scale) with statements that represented the perceived confidence gained during the VHEL sessions. When responding to the following statement, “The VHEL sessions increased my confidence when preparing and presenting a case for CRIBS (Clinical Reasoning in Basic Sciences) course,'' 70% of respondents agreed or strongly agreed. This shows that the majority of respondents were of the opinion they gained confidence in case presentations as part of a required course, integrating learning activities across courses. When responding to the following statement, “I feel less hesitant to speak up in front of others and offer insight after participating in VHEL sessions,” 61% of respondents agreed or strongly agreed. This indicates increased comfort in discussing patient care among a group of colleagues, an essential component of a career in healthcare. Moreover, 78% of respondents agreed or strongly agreed with the statement, “I became more decisive in my clinical decision-making throughout the week of VHEL sessions,” suggesting the progression of VHEL sessions was associated with a perceived incremental improvement in clinical reasoning. When responding to the following statement, “As a result of the VHEL sessions, I am more prepared and confident in conversing with and deducing the condition of standardized patients in my primary skills course,” 76% of respondents agreed or strongly agreed, providing additional evidence of cross-course integration and confidence during interactions when in a simulated hospital setting.

## Discussion

VHEL sessions require complex problem-solving skills and efficient teamwork by medical students. The vast majority of students reported increased perceived confidence in clinical skills and decision-making after the VHEL sessions. More specifically, students reported increased perceived confidence in communicating with and diagnosing their standardized patients in their primary care skills course after participating in VHEL sessions. This provides support to incorporate VHEL into the medical curriculum to maximize and integrate learning across courses. Students also reported they were better able to prioritize critical issues efficiently after the session. They noted an improvement in their ability to order labs, a valuable skill during clerkship years in medical school. Most students agreed that the VHEL was applicable to their clerkship rotations, aligning with the initial goal of creating VHEL during the pandemic as medical students were removed from hospitals. The overall positive feedback suggests not only the benefits of increased perceived confidence in clinical skills but also student engagement across all four years of medical school, thus offering a complementary addition to traditional training before and during clerkship rotations [7]. Moreover, these data support the idea that when pivoting to new modalities for learning, medical students adapt and continue to grow in their clinical skills regardless of the learning environment [8].

Students gain copious amounts of knowledge and skills during the first two didactic years (M1-M2). Although learning rates differ between students, most medical students have a better understanding of general clinical skills as they end their second year relative to their first year. Therefore, we sought to examine any nuances in the perceived total confidence relative to the year in medical school. Differences in perceived total confidence at the end of the VHEL sessions were not statistically significant (p = 0.09) between the M1 and M2 cohorts (Figure 1). However, M2 medical students’ perceived total confidence level score total was somewhat lower than M1s. This may be a result of a higher initial clinical knowledge base and skill set of the M2 relative to M1 students. While we restricted our comparative statistical analysis to differences within the pre-clerkship (M1-M2) experience and clerkship (M3-M4) experience, it is interesting to note M1 students’ perceived total confidence level is above that of M3 and M4 medical students, which may be due to a greater room for clinical skill growth of M1 students (Figure 1) [9]. Thus, there may be potential value in adopting VHEL sessions early in the medical school curriculum to offer the greatest return on investment in developing M1 clinical skills to build upon in subsequent academic years (M2-M4).

The pandemic served as the impetus for simulated VHEL models, among other creative methods; however, medical education is faced with determining which approaches to retain and/or modify in the post-pandemic curriculum. Continuation of simulated models like VHEL may be placed fluidly into medical school curricula, such as incorporation into primary care skills classes or labs. The virtual hospital allows students to learn other aspects of medicine complementary to interprofessional education (IPE) and simulation labs like teamwork, staff management, and continuous patient care. Likewise, the VHEL approach affords perspective into telemedicine and the opportunity to practice building a relationship with patients via an electronic service [8,10,11]. These new skills are critical to current medical practice [9,10]. A vast array of evidence demonstrates that virtual-based simulation training has positively impacted the level of confidence and perceived skills growth for learners in undergraduate medical education [12,13].

This retrospective study had limitations due to the low survey response rate (25%), reducing the power of our study. This is potentially due to the time gap (months) between the VHEL sessions and the collection of the survey responses. Furthermore, VHEL was a required course created for the M3 and M4 years while the VHEL for M1 and M2 students was an elective. Variation existed between the pre-clerkship and clerkship VHEL sessions to ensure level-appropriate content, which may have confounded the total perceived confidence data. While there is no avoiding the need for level-appropriate content, requiring the VHEL sessions for both pre-clerkship and clerkship students will minimize bias due to any differences that may exist in students who elect to participate in extra coursework compared to students required to complete the VHEL course.

To strengthen future analyses of virtual learning interventions, surveys would be administered at the beginning and end of their years in medical school to quantify the impact of the VHEL in the curriculum and assess objective measures of confidence and skill level, rather than self-reported perceptions. The circumstances surrounding the pandemic and the rapidity of educational adaptations, such as this VHEL, limited the time and ability to administer pre-surveys. We recommend survey data collection immediately following the VHEL sessions as part of the course requirements to overcome a low response rate. Additional research may explore the alignment of the virtual hospital simulations alongside instruction covering specific body systems or other aspects of the curriculum.

## Conclusions

For each year of medical school students (M1-M4), the VHEL sessions served a specific purpose. For clerkship students (M3 and M4), it was out of necessity after having been removed from the hospital setting; for M2 students, it was an opportunity to prepare for upcoming clinical rotations; and for M1 students, it was a platform to apply didactic and lab content to a hospital setting. While the driving force and learning level varied across M1 to M4 students, the data indicate the majority of medical student respondents reported an increase in perceived total confidence following completion of the VHEL sessions. While there were no statistically significant differences between perceived total confidence scores between pre-clerkship students (M1-M2) or between clerkship students (M3-M4), M1 respondent data displayed a moderately higher level of perceived total confidence overall, suggesting implementation of VHEL sessions during the spring semester of the first year of medical school may offer the largest gains. Moreover, the data demonstrate confidence gained by medical students was complementary with multiple other courses, such as interprofessional education, primary care skills, standardized patient exercises, and clinical reasoning in basic sciences. Self-reported confidence in a number of highly relevant skills included writing an admission note, formulating differential diagnoses, and ordering appropriate labs. Regardless of the place along the undergraduate medical education continuum, students reported perceived confidence and skill growth following VHEL sessions.
